# Phosphonium-based Ionic Liquid Modified Activated Carbon from Mixed Recyclable Waste for Mercury(II) Uptake

**DOI:** 10.3390/molecules24030570

**Published:** 2019-02-05

**Authors:** Mohamed A. Habila, Zeid A. AlOthman, Ayman A. Ghfar, Maha I.M. Al-Zaben, Ahmed A.S. Alothman, Ahmed A. Abdeltawab, Adel El-Marghany, Mohamed Sheikh

**Affiliations:** 1 Advanced Materials Research Chair, Chemistry Department P. O. Box 2455, College of Science, King Saud University, Riyadh 11451, Saudi Arabia; aghafr@ksu.edu.sa (A.A.G.); moshaik@ksu.edu.sa (M.S.); 2 Department of Chemistry, College of Science, King Saud University, P.O. Box 2455, Riyadh 11451, Saudi Arabia; mzaben@ksu.edu.sa (M.I.M.A.-Z.); aabdelsattar@ksu.edu.sa (A.A.A.); amarghany@ksu.edu.sa (A.E.-M.); 3 Department of Agricultural Engineering, College of Agriculture, King Saud University, Riyadh-11451, Saudi Arabia; Othmana@ksu.edu.sa; 4 Department of Chemistry, College of Science, Suez University, Ismailia 41522, Egypt

**Keywords:** water treatment by adsorption, mercury remediation, ICP-MS, kinetic studies, isotherm models, activated carbon from mixed recyclable waste, phosphonium-based ionic liquid

## Abstract

The contamination of water surfaces by mercury is a dangerous environmental problem due to its toxicity, which leads kidney damage. Activated carbon from mixed recyclable waste modified by phosphonium-based ionic liquid (IL-ACMRW) was therefore prepared and evaluated for Hg(II) remediation. The activated carbon used in this study was prepared from mixed waste, including cardboard, papers and palm wastes as cheap raw materials. The mixed Recyclable Waste Activated Carbon was combined with trihexyl(tetradecyl)phosphonium Bis2,4,4-(trimethylpentyl)phosphinate (Cyphos^®^ IL 104) ionic liquid to form an adsorbent with organic-inorganic content, in order to improve the Hg(II) uptake from aqueous solutions. FTIR confirms the presence of P, C=O and OH after this modification. The adsorption process was investigated and the evaluated results showed that the capacity was 124 mg/g at pH 4, with a contact time of 90 min, an adsorbent dose of 0.4 g/L, and a Hg(II) concentration of 50 mg/L. This Hg(II) adsorption capacity is superior than that reported in the literature for modified multiwall carbon nanotubes. The adsorption of Hg(II) on the modified activated carbon from mixed recyclable waste was found to follow the pseudo second-order kinetics model. Isotherms of adsorption were analyzed via Freundlich and Langmuir models. The results indicated that Freundlich is the best model to describe the process, suggesting multilayer adsorption.

## 1. Introduction

Mercury is reported to cause hazardous effects for human and living organisms due to its toxic effects and non-biodegradable nature, as well as bioaccumulation via the food chain [1,2,3]. It is listed in the pollutants priority by the US EPA. In addition, the EPA assigned the permitted concentration limits for mercury in drinking water to be 0.002 mg/L and for that of discharge waste water to not exceed 10 mg/L [4,5]. The main sources of mercury pollution are the emissions from coal combustion [2,3]. Other sources include different industrial activities in the fields of paper, pigments, oil refinery and pharmaceutical production [6]. There are many methods for mercury(II) remediation from wastewater before their discharge to surface water including precipitation, membrane filtration, ion exchange column and adsorption [7,8]. Liu et al., 2015, tested the mercury removal with two homogeneous photo-fenton-like reactions using photochemical reactors [9]. Chiarle et al., 2000, applied ion exchange resin to adsorb mercury and evaluate the process using batch experiments using Duolite GT-73 [10]. Among various methods for mercury removal, adsorption has been shown to be more efficient than other water purification methods [11,12]. Johari et al., 2014, have tested mercury(II) remediation using different adsorbents such as silica gel and sulfur-functionalised silica gel, and they reported adsorption capacities of 40.95, 93.32 and 102.37 mg/g, for silica gel prepared by tetraethyl orthosilicate, silica gel prepared with bis(triethoxysilylpropyl) tetrasulfide and silica gel prepared by 3-mercaptopropyl trimethoxysilane, respectively [13]. Recently, considerable efforts have been directed to use low-cost adsorbents for the removal of pollutants. These adsorbents include agricultural waste, coal, sludge and activated carbon [14].

Adsorbents based on activated carbons showed a high efficiency in the removal of various pollutants and there are many studies concentrating on their preparation from low cost alternative sources like agricultural waste [12,15]. The application of activated carbon for adsorptive removal is affected by their chemical properties and porous structures [16]. For example, Zhang et al., 2005, developed different kinds of activated carbons for Hg removal using sewage sludge as raw materials and different activating agents such as H_2_SO_4_, H_3_PO_4_ and ZnCl_2_ [17]. Their results indicated that the quality and efficiency of carbon is significantly dependent on the activation process, and they showed that ZnCl_2_ is superior in developing efficient carbon. Vernon and Bonzongo, 2014, have studied the adsorption of mercury onto iron particles by Batch techniques [18]. Krishnan and Anirudhan, 2002, studied mercury(II) remediation by adsorption carbon activated with steam and with sulphurised steam using bagasse as starting raw materials [19]. They reported that the rate of the adsorption process followed the pseudo-second-order kinetic model [19]. Yardim et al., 2003, investigated mercury(II) remediation using activated carbon prepared from furfural by polymerization followed by carbonization and final activation with steam [20].

The modification of carbon nanotubes has been investigated in the literature to improve their mercury(II) adsorption efficiency. For example, Gupta and Vidyarthi, 2014, prepared sulfur incorporated MWCNT (CNT-S), which showed a high adsorption capacity compared with original carbon nanotubes [21]. Hadavifar et al., 2014, investigated thiolated multi-walled carbon nanotube MWCNTs for mercury(II) adsorption and successfully applied them for waste water treatment [22]. Zhang et al., 2014, tested thiol-functionalized multiwall carbon nanotube-Fe3O4 nanocomposites for mercury removal, which showed an adsorption capacity of 65 mg/g [23]. Chem et al., 2014, successfully applied modified multi-walled carbon nanotubes for mercury removal [24] Ganzagh et al., 2016, applied Ag supported on nanomesoporous silica supported silica and recommended them for the purification of mercury(II) from water solutions [25]. Recently, ionic liquids have been applied to improve the adsorption efficiency. Qureshi et al., 2012, introduced ionic liquid supported XAD-4 resin as an efficient sorbent for water treatment applications [26]. The ionic liquids act as environmental friendly solvents which facilitate the road for green chemistry. The ionic liquids have melting point less than 100 °C, are nonflammable, have a negligible low vapour pressure, and have a high stability both chemically and thermally. In addition, ionic liquids can be manipulated with various structures by controlling the cation and anion, leading to highly promised solvents for separation and extraction [27,28]. Therefore, this work aims to achieve mercury(II) remediation using activated carbon from mixed recyclable waste modified by a phosphonium—based ionic liquid. For this purpose, activated carbon from mixed recyclable waste is combined with Trihexyl(tetradecyl)phosphonium Bis2,4,4-(trimethylpentyl) phosphinate (Cyphos^®^ IL 104) ionic liquid by an impregnation process. The modified adsorbent was characterized by SEM and FTIR. Kinetic and equilibrium studies were conducted to evaluate the adsorptive removal process of Hg(II) onto the phosphonium-based ionic liquid modified activated carbon from mixed recyclable waste.

## 2. Results and Discussion

### 2.1. Characterization of the Modified Activated Carbon

**Figure 1:**
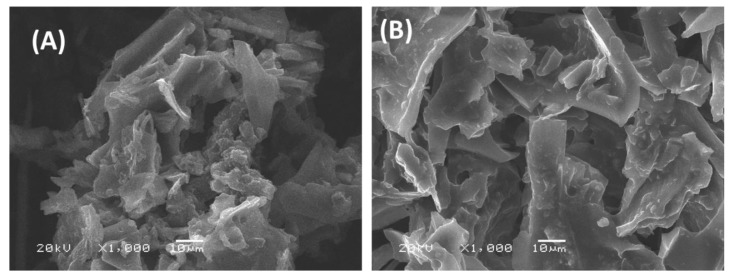
(**A**) SEM images of activated carbon from mixed recyclable waste (ACMRW) and (**B**) the trihexyl(tetradecyl)phosphonium Bis2,4,4-(trimethylpentyl)phosphinate modified activated carbon (IL-ACMRW—Ionic liquid modified activated carbon from mixed recyclable waste).

In this study the activated carbon prepared from mixed recyclable waste (ACMRW) [15] was modified by combination with trihexyl(tetradecyl)phosphonium Bis2,4,4-(trimethylpentyl) phosphinate (Cyphos^®^ IL 104) in order to enhance the surface charge and to increase the tendency to adsorb Hg(II). The surface morphology of the activated carbon prepared from mixed waste and the modified version (IL-ACMRW) were both examined by scanning electron microscopy. Results presented in Figure 1A,B reveal the rough and porous surfaces of both ACMRW and IL-ACMRW. In addition, the structure of the carbon appears as parallel and perpendicular sheets, and includes holes and pores in-between, for both ACMRW and IL-ACMRW.

**Figure 2:**
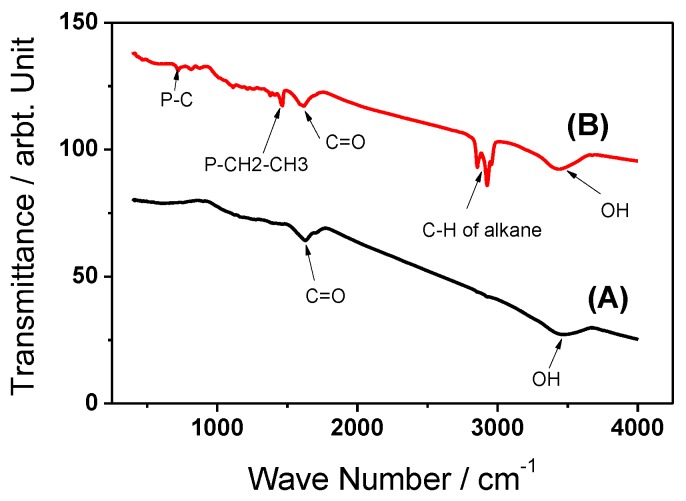
(**A**) FTIR spectra of Activated Carbon from mixed recyclable waste (ACMRW) and (**B**) the trihexyl(tetradecyl)phosphonium Bis2,4,4-(trimethylpentyl)phosphinate modified activated carbon (IL-ACMRW).

In addition, FTIR was applied to identify the surface functional groups of activated carbon from mixed recyclable waste (ACMRW) (Figure 2A) and the trihexyl(tetradecyl)phosphonium Bis2,4,4-(trimethylpentyl)phosphinate modified activated carbon (IL-ACMRW) (Figure 2B). The results show that activated carbon from mixed recyclable waste has limited peaks related to OH at 3457 cm^−1^ and for carbonyl groups at 1636 cm^−1^ (Figure 2A). While in the case of the modified activated carbon (IL-ACMRW) the peak related to OH was detected at 3442 cm^−1^, the carbonyl group was detected at 1626 cm^−1^. C-H of alkane showed clear peaks at 2849 cm^−1^ and 2932 cm^−1^. The peak at 1440 cm^−1^ is related to P-CH_2_-CH_3_, while the medium peak at 750 is related to P-C (Figure 2B). The presence of the phosphorous atom in the framework of the prepared adsorbent is expected to enhance the ability for efficient interaction with Hg ions, in addition to the attraction with carbonyl and hydroxyl groups.

### 2.2. Adsorption Studies

#### 2.2.1. Effect of pH on the Hg(II) Removal

The removal efficiency of heavy metals is reported to be dependent on the pH of the metal solution medium [12,29]. The effect of the pH was evaluated for Hg(II) removal using trihexyl(tetradecyl)phosphonium Bis2,4,4-(trimethylpentyl)phosphinate modified activated carbon from mixed recyclable waste (IL-ACMRW), compared with original activated carbon from mixed recyclable waste (ACMRW). The adsorption capacity was calculated using the following equation, Equation (1):

qe = (C_0_ − C_e_) *v*/*w* (1)

where qe is the adsorption capacity (mg/g), C0 is the initial concentration of Hg(II), Ce is the equilibrium concentration of the Hg(II), *v* is the volume of the solution (mL) and *w* is the weight in gm.

**Figure 3:**
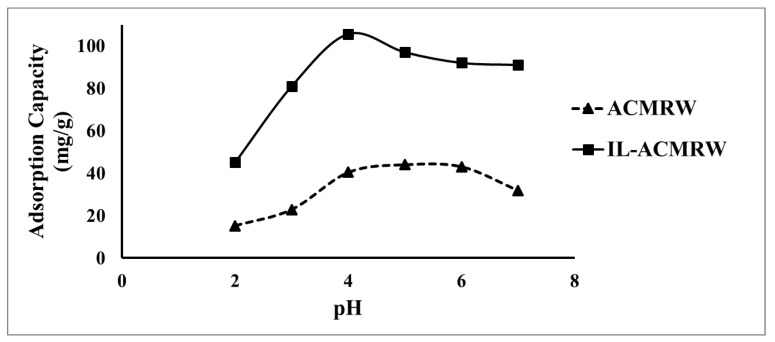
Effect of pH on the adsorption capacity of Hg(II), adsorbent dose 0.5 m/L, Hg(II) solution dose of 120 mg/L. ACMRW- activated carbon from mixed recyclable waste; IL-ACMRW—Ionic liquid modified activated carbon from mixed recyclable waste.

Figure 3 presents the adsorption capacity at pHs ranging from 2 to 7 for IL-ACMRW and ACMRW. The IL-ACMRW showed improved efficiency for the Hg(II) uptake (105 mg/g), while the ACMRW exhibited 44 mg/g. The maximum adsorption capacity was obtained around pH 4. This noticed increase in the adsorption capacity for the uptake of Hg(II) from the aqueous solution is attributed to the presence of phosphorus in the structure of the IL-ACMRW, which enhanced the coordination with Hg(II).

#### 2.2.2. Effect of Contact Time

**Figure 4:**
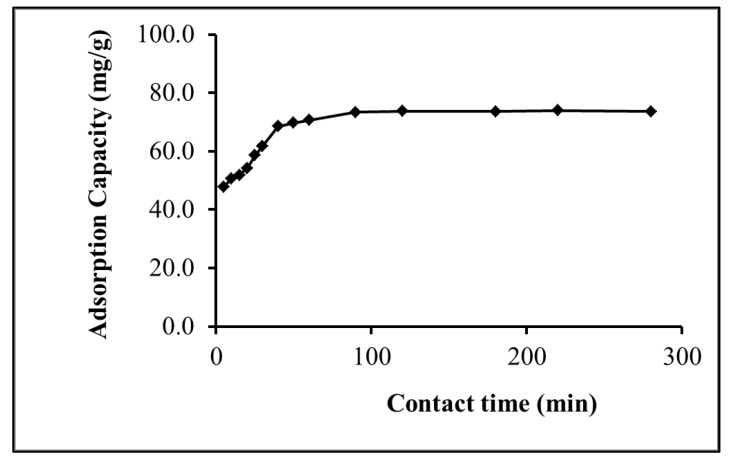
Effect of contact time on Hg(II) adsorption by IL-ACMRW (ionic liquid modified activated carbon from mixed recyclable waste), adsorbent dose 0.5 m/L, Hg(II) solution dose of 20 mg/L at pH 4.

The tendency of IL-ACMRW to adsorb Hg(II) is dependent on the surface active sites which control the rate of adsorption of Hg(II). To investigate this effect, the contact time between the IL-ACMRW and Hg(II) was tested by a time intervals evaluation. This showed that the absorption of Hg(II) is directly proportional with the increase of the time of contact until reaching 90 min, after which no significant change in adsorption capacity was noticed (Figure 4). 90 min is therefore considered to be the equilibrium point (adsorption is equal to that of desorption) [11]. This adsorption behavior trend is in agreement with that reported by Alothman et al. [29] for the adsorption of Cd(II) onto modified agricultural waste.

#### 2.2.3. Kinetic Studies

For a further optimized evaluation of the adsorption process of Hg(II) onto IL-LWAC, different kinetic models were applied. These included the pseudo-first-order equation of Lagergren and the pseudo-second-order kinetic rate equation [30,31].

The equation of the pseudo-first-order equation is Equation (2):

dqt/dt = k_1_(qe − qt) (2)

where qe is the capacity for adsorption of Hg(II) at equilibrium and qt is the capacity of adsorption of Hg(II) at time t; k_1_ is the rate constant of the pseudo-first-order reaction (min^−1^).

When applying the equation in integrated form with qt = 0 to qt = qt, at t = 0 to t = t, the integrated form of Equation (2) becomes

log(qe − qt) = log qe − k_1_t/2.303 (3)

**Figure 5:**
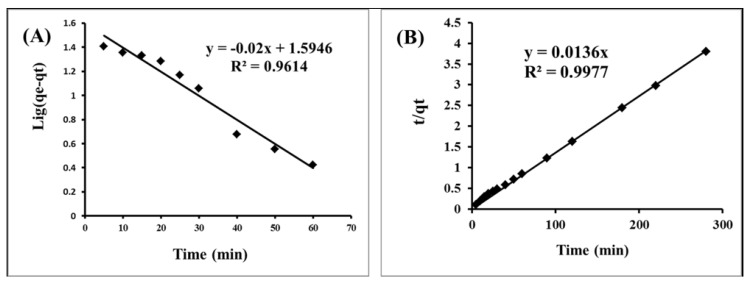
(**A**) Pseudo-first-order and (**B**) pseudo-second-order of the Hg(II) adsorption by IL-ACMRW (ionic liquid modified activated carbon from mixed recyclable waste), adsorbent dose 0.5 m/L, Hg(II) solution dose of 20 mg/L at pH 4.

By setting the data in a graph between log (qe − q) and t, the constants for the pseudo-first-order can be calculated (Figure 5). The obtained k1 values and the linear correlation coefficient are defined in Table 1. It was found that R12 was 0.96 and the calculated qe was 39.3 for the Hg(II) uptake, which is in suitable agreement with the experimental qe. From this result, it can be derived that the model of first order can be applied to explain the adsorption kinetics of Hg(II) onto L-LWAC.

For the further investigation of the kinetic behavior, a second model of the pseudo-second-order is tested using the present adsorption data for Hg(II) onto L-LWAC, as in Equation (4) [32]:

dqt/dt = k2·(qe − qt)^2^ (4)

where qe is the capacity for adsorption of Hg(II) at equilibrium and qt is the capacity of adsorption of Hg(II) at time t. k2 is the rate constant of the pseudo-second-order (g/(mg·min)).

By integration, equation 4 becomes:

t/qt = 1/Kqe^2^ + (1/qe) t (5)

where t is the contact time (min), and qe (mg/g) and q2 (mg/g) are the amount of solute adsorbed at equilibrium. Figure 5 shows the linear relationship of the graph plot of t/qt versus t, from which qe and k can be determined from the slope and intercept, respectively.

The calculated k2 values and corresponding linear regression correlation coefficient values are shown in Table 2, which reveals that this model is suitable for describing the Hg(II) uptake, along with the calculated qe according to this equation, for the Hg(II) uptake is in agreement with the experimental qe. Therefore, it can be reported that there is a migration of the Hg(II) from the solution and that the transfer to the surface of the IL-ACMRW is the rate determining step; this is followed by the migration of the Hg(II) inside the porosity of the IL-ACMRW. Finally, the adsorption of the Hg(II) inside the pores is the more dominant mechanism [33].

#### 2.2.4. Effect of Adsorbent Dosage

**Figure 6:**
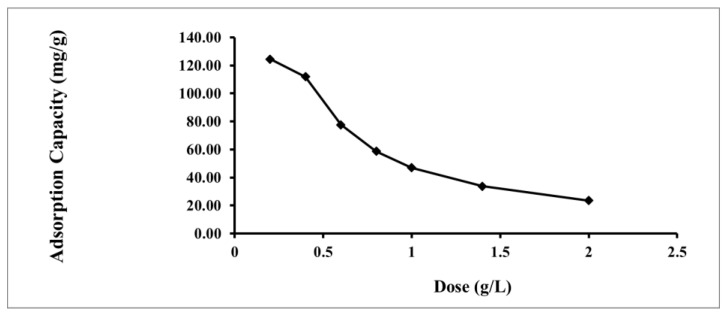
Effect of adsorbent dose on Hg(II) adsorption; Hg(II) solution dose of 50 mg/L at pH 4.

The quantity Hg(II) adsorbed onto IL-ACMRW varied with the varying of the dosages. As shown in Figure 6, the adsorption capacity of Hg(II) decreased from 124 to 23.5 mg/g with an increase in the IL-ACMRW concentration from 0.2 to 2 g/L for the initial Hg(II) concentration of 50 mg/L. On the other hand, the value of the amount of Hg(II) adsorbed increases by using more adsorbents. The increase in the adsorption of Hg(II) is due to the increasing adsorbent amount. This behavior is commonly noticed during adsorption studies [13].

#### 2.2.5. Adsorption Isotherms

**Langmuir Isotherm**

The Langmuir equation was applied to the equilibrium adsorption data for Hg(II) ions onto IL-ACMRW. The Langmuir treatment is built on the basis that the maximum adsorption is due to a saturated monolayer coverage of adsorbate molecules onto the adsorbent surface, that the energy of adsorption is constant and that there is no transmigration of adsorbate to the plane surface [34]

Ce/qe = 1/(q_max_ b) + Ce/qmax (6)

where Ce is the concentration of Hg(II) at equilibrium, and qe is the capacity of the prepared IL-ACMRW to adsorb Hg(II) (mg/g). The qmax is the maximum adsorption capacity (mg/g), assumed by Langmuir. The b constant is the adsorption energy. The Langmuir equilibrium constant, KL, is equal to

KL = q_max_ b (7)

**Figure 7:**
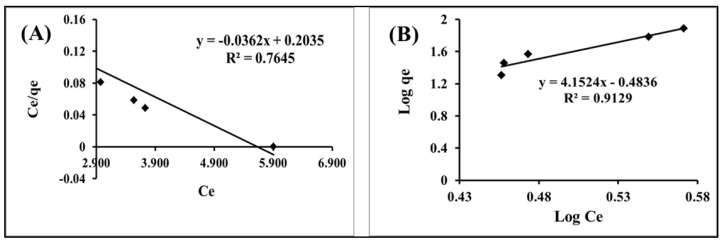
(**A**) Langmuir adsorption isotherms and (**B)** Freundlich adsorption isotherm of Hg(II) ions onto IL-ACMRW (ionic liquid modified activated carbon from mixed recyclable waste).

This study’s data for Hg(II) uptakes onto IL-ACMRW was applied for the Langmuir assumption, and Figure 7 presents this graph. The graph shows that the value of R2 was 0.764, which can be considered to be a bad correlation. Therefore, the Langmuir assumption is not suitable to fit Hg(II) uptakes onto IL-ACMRW.

**Freundlich isotherm**

logqe = logK + 1/n logCe(8)

where Ce is the concentration of Hg(II) (mg/L) at equilibrium and qe the quantity of Hg(II) taken onto IL-ACMRW (mg/g) at equilibrium. The KF refers to the Freundlich assumption for adsorbent capacity, while n refers to the favorable nature of the process of the Hg(II) uptake onto IL-ACMRW. The plotting of log qe & log Ce will have the slope equal to 1/n and the intercept equal to logKF (Figure 7).

The constants calculated for Langmuir and Freundlich are shown in Table 2. The process of Hg(II) uptakes onto IL-ACMRW was well correlated with the Freundlich equation (R^2^ = 0.912), while in the case of Langmuir the correlation (R^2^ = 0.764) did not achieve the same degree of matching that Freundlich did. This suggests different behaviors during the adsorption process, which may be due to the presence of heterogeneous surfaces in the prepared IL-ACMRW, due to a combination of ionic liquid with the activated carbon from mixed recyclable waste [27]. In addition, this confirms the presence of energy variation within the heterogeneous surfaces of IL-ACMRW [35,36].

Some of the previously published studies [30,31,32,33,34] related to Hg(II) removal are compared with the present study, as shown in Table 3. The comparison reveals that the capacity for Hg(II) removal onto trihexyl(tetradecyl)phosphonium Bis2,4,4-(trimethylpentyl)phosphinate modified activated carbon from mixed recyclable waste (IL-ACMRW) is better than that of thiolated multiwall carbon nanotubes [31], thiol-functionalized superparamagnetic carbon nanotubes [32], and Ag supported on nanomesoporous silica [34]. Meanwhile, the capacity was lower than that of sulfur incorporated multiwall carbon nanotubes [30]. In addition, this comparison confirms the valuable applications for water purification and saving the environment of carbon from low cost sources such as the one described in this study, trihexyl(tetradecyl)phosphonium Bis2,4,4-(trimethylpentyl)phosphinate modified activated carbon from mixed recyclable waste (IL-ACMRW).

## 3. Materials and Methods

### 3.1. Materials and Reagents

We used chemicals and reagents with high-purity for the solution preparation. Phosphonium-based ionic liquid: trihexyl(tetradecyl)phosphonium Bis2,4,4-(trimethylpentyl)phosphinate (Cyphos^®^ IL 104) was obtained from Cytec Industries Inc., New Jersey, NJ, USA. Stock solutions of mercury ions with a concentration of 1000 mg/L (Sigma, St. Louis, MO, USA) were diluted day by day to obtain working solutions. Activated carbon from mixed recyclable waste was prepared from solid wastes including cardboard, papers and palm wastes. This activated carbon has been characterized and reported in the literature [15,37].

### 3.2. Preparation of Trihexyl(tetradecyl)phosphonium Bis2,4,4-(trimethylpentyl) Phosphinate (Cyphos^®^ IL 104) Modified Activated Carbon (IL-ACMRW)

The modification of the activated carbon from mixed recyclable waste was done by impregnation, as described in the literature, but with some modifications [37,38]. In detail, activated carbon (5 g) was added to 200 mL of 0.25% (*m*/*v*) trihexyl(tetradecyl) phosphonium Bis2,4,4-(trimethylpentyl)phosphinate (Cyphos^®^ IL 104) solution in acetone, and the mixture was stirred for 24 h. Then, the product was isolated by filtration, before the adsorbent was cleaned with deionized water washing and drying at 100 °C.

### 3.3. Adsorption Studies of Hg(II) Onto IL-ACMRW

The prepared IL-ACMRW or ACMRW, (0.05 g) was weighed and placed in 100 mL flasks and then 50 mL of Hg(II) solution was added. The flask was placed on a shaker and kept at 150 rpm for 2 h, at a temperature of 25 °C. Finally, the solution was separated from the adsorbent by filtration, and the Hg(II) was measured by ICP-MS. The blank replicates were included without adding the adsorbent. The adsorption capacity of the IL-ACMRW samples was calculated using equation 1.

## 4. Conclusion

The combination of activated carbon from mixed recyclable waste (ACMRW) with trihexyl(tetradecyl) phosphonium Bis2,4,4-(trimethylpentyl) phosphinate (Cyphos^®^ IL 104) has improved the adsorption capacity. The capacity of IL-ACMRW for Hg(II) uptake was about 124 mg/g. This was tested at pH 4, with an adsorbent dose of 0.2 g/L, a Hg(II) solution dose of 50 mg/L and a contact time of 90 min. The experiments for kinetic investigations have shown that the process of Hg(II) uptake onto IL-ACMRW is fits well with the pseudo-second model. Meanwhile, the applications of Langmuir and Freundlich assumptions for adsorption isotherms reveal that the Freundlich model is more suitable for a description of the Hg(II) uptake onto IL-ACMRW. This confirms the heterogeneous properties of the IL-ACMRW, which could be related to the nature of the mixed waste origin of the activated carbon used in this work. In addition to this, it confirms the successful combination of ionic liquid which improved mercury removal by adsorption.

## Figures and Tables

**Table 1:** Kinetic constants for Hg(II) adsorption onto IL-ACMRW—Ionic liquid modified activated carbon from mixed recyclable waste.

The Pseudo-First-Order			The Pseudo-Second-Order		
Rate	qe (mg/g)	Correlation	Rate	qe (mg/g)	Correlation
constant		coefficient	constant		coefficient
(K_1_)		(R^2^_1_)	(K_2_)		(R^2^_2_)
0.046	39.3	0.96	-	73.5	0.99

**Table 2:** Langmuir and Freundlich constants of Hg(II) ions on IL-ACMRW (ionic liquid modified activated carbon from mixed recyclable waste).

**Langmuir Constants**	**Constant**	**In the case of Hg(II) adsorption onto IL-ACMRW**
	K_L_	4.885
	b	0.177
	Q_max_	27.6
	R^2^	0.764
**Freundlich Constants**	K_F_	0.32
	n	0.24
	R^2^	0.912

**Table 3:** Comparison of the ability of various adsorbent for Hg(II) uptake with IL-ACMRW (ionic liquid modified activated carbon from mixed recyclable waste).

Adsorbent	q_max_ (mg/g)	Reference
Trihexyl(tetradecyl)phosphonium Bis2,4,4-(trimethylpentyl)phosphinate modified activated carbon from mixed recyclable waste (IL-ACMRW)	124	Current work
Sulfur incorporated multiwall carbon nanotube	151.5	[21]
Thiolated multiwall carbon nanotube	84.66	[22]
Thiol-functionalized superparamagnetic carbon nanotubes	65.52	[23]
Carboxylic modified multiwall carbon nanotube	127.6	[24]
Ag supported on nanomesoporous silica	42.26	[25]

## References

[1] Byun Y., Koh D.J., Shin D.N. (2011). Removal mechanism of elemental mercury by using non-thermal plasma. Chemosphere.

[2] Hanlon J. (2007). Analytical methods for mercury in national pollutant discharge elimination systems (npdes) permits. O.o.W. Management. US EPA.

[3] Yang S., Yan N., Guo Y., Wu D., He H., Qu Z., Li J., Zhou Q., Jia J. (2011). Gaseous Elemental Mercury Capture from Flue Gas Using Magnetic Nanosized (Fe_3−x_Mn_x_)_1−δ_O_4_. Environ. Sci. Technol..

[4] Nam K.H., Gomez-Salazar S., Tavlarides L.L. (2003). Mercury(II) Adsorption from Wastewaters Using a Thiol Functional Adsorbent. Ind. Eng. Chem. Res..

[5] (2001). National primary drinking water standards. Report EPA 816-F-01-007, Washington DC. USEPA.

[6] Luo C.-S., Huang S.-D. (1993). Removal of Copper from Aqueous Amminecopper(II) Solution by Foam Flotation. Sep. Sci. Technol..

[7] Henneberry Y.K., Kraus T.E.C., Fleck J.A., Krabbenhoft D.P., Bachand P.M., Horwath W.R. (2011). Removal of inorganic mercury and methylmercury from surface waters following coagulation of dissolved organic matter with metal-based salts. Sci. Total Environ..

[8] Kurniawan T.A., Chan G.Y.S., Lo W.-H., Babel S. (2006). Physico–chemical treatment techniques for wastewater laden with heavy metals. Chem. Eng. J..

[9] Liu Y., Zhang J., Yin Y. (2015). Removal of Hg0 from flue gas using two homogeneous photo-fenton-like reactions. AIChE J..

[10] Chiarle S., Ratto M., Rovatti M. (2000). Mercury removal from water by ion exchange resins adsorption. Water Res..

[11] Al Othman Z.A., Habila M.A., Hashem A. (2013). Removal of zinc(II) from aqueous solutions using modified agricultural wastes: kinetics and equilibrium studies. Arabian J. Geosci..

[12] AlOthman Z.A., Habila M.A., Ali R., Abdel Ghafar A., El-din Hassouna M.S. (2014). Valorization of two waste streams into activated carbon and studying its adsorption kinetics, equilibrium isotherms and thermodynamics for methylene blue removal. Arabian J. Chem..

[13] Johari K., Saman N., Mat H. (2013). A comparative evaluation of mercury(II) adsorption equilibrium and kinetics onto silica gel and sulfur-functionalised silica gels adsorbents. The Canadian J. Chem. Eng..

[14] Bailey S.E., Olin T.J., Bricka R.M., Adrian D.D. (1999). A review of potentially low-cost sorbents for heavy metals. Water Res..

[15] Habila M.A., Alothman Z.A., Ali R., Ghafar A.A., Hassouna M.S.E.-D. (2013). Removal of Tartrazine Dye onto Mixed-Waste Activated Carbon: Kinetic and Thermodynamic Studies. CLEAN—Soil Air Water.

[16] Aworn A., Thiravetyan P., Nakbanpote W. (2008). Preparation and characteristics of agricultural Recyclable Waste Activated Carbon by physical activation having micro- and mesopores. J. Anal. Appl. Pyrol..

[17] Zhang F.-S., Nriagu J.O., Itoh H. (2005). Mercury removal from water using activated carbons derived from organic sewage sludge. Water Res..

[18] Vernon J.D., Bonzongo J.-C.J. (2014). Volatilization and sorption of dissolved mercury by metallic iron of different particle sizes: Implications for treatment of mercury contaminated water effluents. J. Hazard. Mater..

[19] Anoop Krishnan K., Anirudhan T.S. (2002). Removal of mercury(II) from aqueous solutions and chlor-alkali industry effluent by steam activated and sulphurised activated carbons prepared from bagasse pith: kinetics and equilibrium studies. J. Hazard. Mater..

[20] Yardim M.F., Budinova T., Ekinci E., Petrov N., Razvigorova M., Minkova V. (2003). Removal of mercury (II) from aqueous solution by activated carbon obtained from furfural. Chemosphere.

[21] Gupta A., Vidyarthi S.R., Sankararamakrishnan N. (2014). Enhanced sorption of mercury from compact fluorescent bulbs and contaminated water streams using functionalized multiwalled carbon nanotubes. J. Hazard. Mater..

[22] Hadavifar M., Bahramifar N., Younesi H., Li Q. (2014). Adsorption of mercury ions from synthetic and real wastewater aqueous solution by functionalized multi-walled carbon nanotube with both amino and thiolated groups. Chem. Eng. J..

[23] Zhang C., Sui J., Li J., Tang Y., Cai W. (2012). Efficient removal of heavy metal ions by thiol-functionalized superparamagnetic carbon nanotubes. Chem. Eng. J..

[24] Chen P.H., Hsu C.-F., Tsai D.D.-W., Lu Y.-M., Huang W.-J. (2014). Adsorption of mercury from water by modified multi-walled carbon nanotubes: adsorption behaviour and interference resistance by coexisting anions. Environ. Technol..

[25] Ganzagh M.A.A., Yousefpour M., Taherian Z. (2016). The removal of mercury(II) from water by Ag supported on nanomesoporous silica. J. Chem. Biol..

[26] Qureshi U.A., Solangi A.R., Memon S.Q., Taqvi S.I.H., Memon N. (2012). Ionic Liquid Modified Resin for the Adsorptive Removal of Dibutyl Phthalate: Equilibrium, Kinetic, and Thermodynamic Studies. CLEAN—Soil Air Water.

[27] Rogers R.D., Seddon K.R. (2003). Chemistry. Ionic liquids—Solvents of the uture?. Science.

[28] Bosmann A., Datsevich L., Jess A., Lauter A., Schmitz C., Wasserscheid P. (2001). Deep desulfurization of diesel fuel by extraction with ionic liquids. Chem. Commun..

[29] Al Othman Z.A., Hashem A., Habila M.A. (2011). Kinetic, Equilibrium and Thermodynamic Studies of Cadmium(II) Adsorption by Modified Agricultural Wastes. Molecules.

[30] Yuh-Shan H. (2004). Citation review of Lagergren kinetic rate equation on adsorption reactions. Scientometrics.

[31] Ho Y.S., McKay G. (2009). The kinetics of sorption of basic dyes from aqueous solution by sphagnum moss peat. The Canadian J. Chem. Eng..

[32] El-Toni A.M., Habila M.A., Ibrahim M.A., Labis J.P., AlOthman Z.A. (2014). Simple and facile synthesis of amino functionalized hollow core-mesoporous shell silica spheres using anionic surfactant for Pb(II), Cd(II), and Zn(II) adsorption and recovery. Chem. Eng. J..

[33] Langmuir I. (1918). The adsorption of gases on plane surfaces of glass, mica and platinum. J. Am. Chem. Soc..

[34] Hutson N.D., Yang R.T. (1997). Theoretical basis for the Dubinin-Radushkevitch (D-R) adsorption isotherm equation. Adsorption.

[35] Freundlich H. (1906). Uber Die Adsorption in Lösungen. J. Phys. Chem..

[36] AlOmar M.K., Alsaadi M.A., Jassam T.M., Akib S., Ali Hashim M. (2017). Novel deep eutectic solvent-functionalized carbon nanotubes adsorbent for mercury removal from water. J. Colloid Interf. Sci..

[37] Habila M., Yilmaz E., Alothman Z.A., Soylak M. (2014). Flame atomic absorption spectrometric determination of Cd, Pb, and Cu in food samples after pre-concentration using 4-(2-thiazolylazo) resorcinol-modified activated carbon. J. Ind. Eng. Chem..

[38] Yusuf N.Y., Masdar M.S., Isahak W.N.R.W., Nordin D., Husaini T., Majlan E.H., Rejab S.A.M., Chew C.L. Ionic liquid-impregnated activated carbon for biohydrogen purification in an adsorption unit. Presented at the 29th Symposium of Malaysian Chemical Engineers (SOMChE).

